# Correction: An essential role for α4A-tubulin in platelet biogenesis

**DOI:** 10.26508/lsa.202101132

**Published:** 2021-06-21

**Authors:** Catherine Strassel, Maria M Magiera, Arnaud Dupuis, Morgane Batzenschlager, Agnès Hovasse, Irina Pleines, Paul Guéguen, Anita Eckly, Sylvie Moog, Léa Mallo, Quentin Kimmerlin, Stéphane Chappaz, Jean-Marc Strub, Natarajan Kathiresan, Henri de la Salle, Alain Van Dorsselaer, Claude Ferec, Jean-Yves Py, Christian Gachet, Christine Schaeffer-Reiss, Benjamin T Kile, Carsten Janke, François Lanza

**Affiliations:** 1 Université de Strasbourg, Institut National de la Santé et de la Recherche Médicale, Etablissement Français du Sang Grand Est, Unité Mixte de Recherche-S 1255, Fédération de Médecine Translationnelle de Strasbourg, Strasbourg, France; 2 Institut Curie, Paris-Sciences-et-Lettres Research University, CNRS UMR3348, Orsay, France; 3 Université Paris Sud, Université Paris-Saclay, CNRS UMR3348, Orsay, France; 4 Laboratoire de Spectrométrie de Masse BioOrganique, Institut Pluridisciplinaire Hubert Curien, CNRS UMR7178, Université de Strasbourg, Strasbourg, France; 5 ACRF Australian Cancer Research Foundation Chemical Biology Division, the Walter and Eliza Hall Institute of Medical Research, Parkville, Australia; 6 Anatomy and Developmental Biology, Monash Biomedicine Discovery Institute, Monash University, Melbourne, Australia; 7 Laboratoire de Génétique Moléculaire et d’Histocompatibilité, Centre Hospitalier Régional et Universitaire Morvan, INSERM U1078, EFS Bretagne, Brest, France; 8 EFS Centre-Pays de la Loire, site d’Orléans, France

Published online 13 February 2019. DOI 10.26508/lsa.201900309.

In the original version of this article, a factual error occurred in Fig 4B. The blot corresponding to TUBA was mistakenly duplicated from that for TUBA4A. This has now been corrected in a new version of the figure. This does not affect the validity of our conclusion that “… that the overall α-tubulin levels are unaltered…” (page 6 second paragraph).

The authors apologize for this error.

**Figure:**
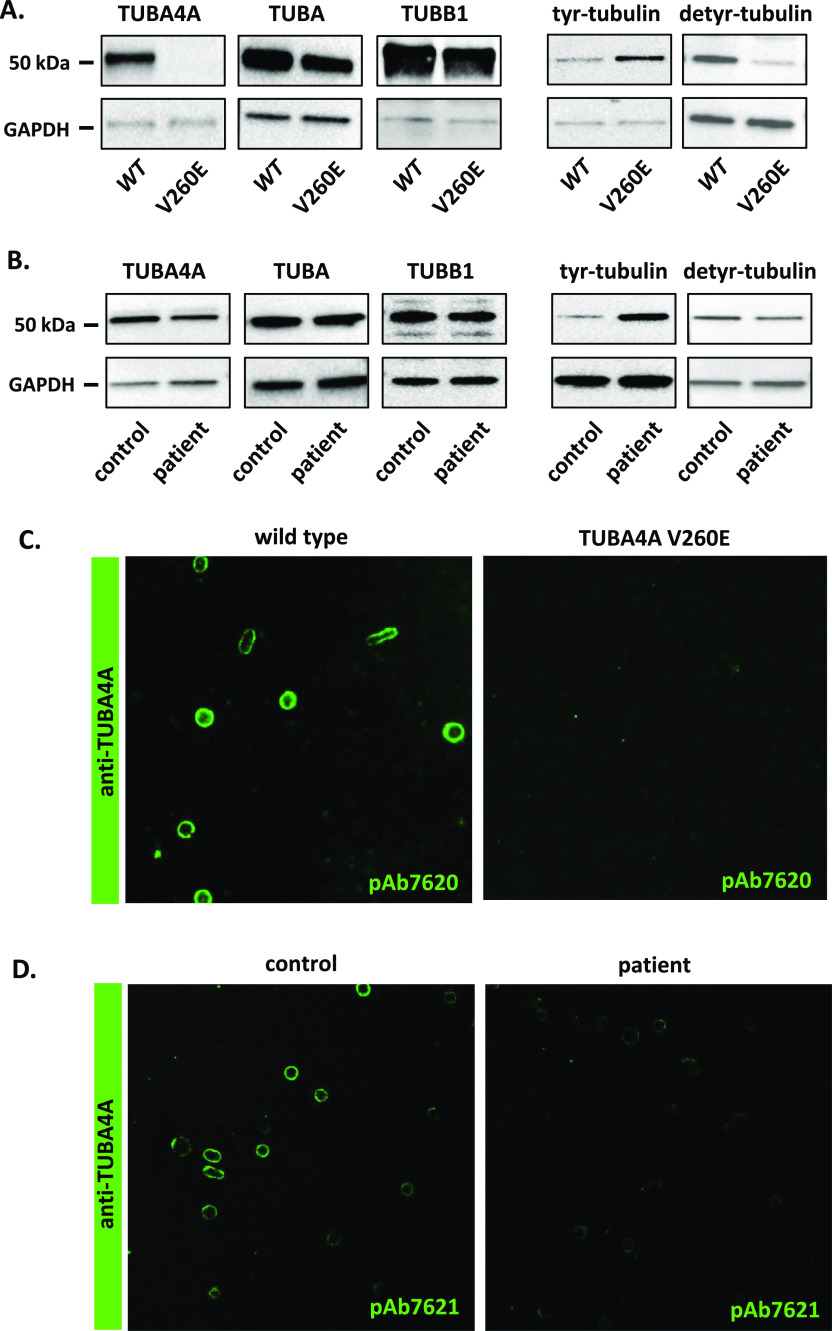


## Supplementary Material

Reviewer comments

