# Supplementary material for: An essential role for α4A-tubulin in platelet biogenesis
Source: Life Sci Alliance. 2019 Feb 13;2(1):e201900309. doi: 10.26508/lsa.201900309 (PMC6374996; doi:10.26508/lsa.201900309)
Supplement: Supplementary file 1 [file LSA-2019-00309_Supplemental_Experimental_Procedures.pdf]

Supplemental Experimental Procedures:

Mice: The protocols carried out in this study were in accordance and approved by the CREMEAS Committee on the Ethics of Animal Experiments of the University of Strasbourg (Comité Régional d'Ethique en Matière d'Expérimentation Animale Strasbourg). Permit Number 2016-121 414 599 992.

Purification of tubulin from human platelets*:* Platelets were isolated from fresh ACD-anticoagulated blood, and washed once in PEM buffer (100 mM Pipes, 10 mM EDTA, 2 mM $MgCl_2$, pH 6.9) supplemented with 1 nM PGI_2. Following centrifugation the platelet pellet was resuspended in cold PEM containing 2 mM GTP and lysed by sonication on ice. Repeated cycles of depolymerization at 0°C and polymerization at 37°C in the presence of 4 M glycerol and 1 mM GTP were then performed, with separation of the polymerized form by ultracentrifugation at 37°C. Care was taken to eliminate actin, which is the main contaminant in these procedures. Following two cycles of depolymerization-polymerization we usually obtained a minimum of 150 µg of tubulin with >90% purity from a 400-mL blood donation.

Platelet washing procedure: Platelets were washed according to Cazenave et al.[24].

RT-PCR amplification of tubulin transcripts in cultured MKs : RNA was extracted from $CD34^+$ cells (day 0) and at different time points of culture (days 4, 7, 10 and 12) using an RNeasy® Mini kit (QIAGEN) following manufacturer's instructions. RNA was subjected to RT-PCR with primers specific for TUBA1, TUBA4A, TUBA8, and TUBB1 (primer sequences in Table S1). Amplified bands (30 cycles, extension at 57°C) were separated by agarose gel electrophoresis and their intensity was measured using GelDoc EZ System. Expression levels were normalized to that of 18S.

LC-MS/MS analysis of platelet tubulin: Tryptic peptides were analyzed on a nanoACQUITY Ultra-Performance-LC system (UPLC, Waters, Milford, USA) coupled to a Q-TOF mass spectrometer (maXis 4G, Bruker Daltonics, Bremen, Germany) equipped with a nano electrospray source operating in positive mode. The UPLC system was equipped with a Symmetry C18 precolumn (20×0.18 mm, 5 µm particle size) and an ACQUITY UPLC® BEH130 C18 separation column (75 µm×250 mm, 1.7 µm particle size, Waters). Raw data collected during nanoLC-MS/MS analyses were processed, and then converted into mgf files with DataAnalysis (BrukerDaltonics, Bremen Germany). The MS/MS data were analyzed using the MASCOT 2.4.1 algorithm (Matrix Science, London, UK) and results were loaded

into the Scaffold software (version 3.6.5, Proteome Software Inc., Portland, USA) and filtered in order to set the false discovery rate to less than 1%.

LC-SRM analyses of platelet α-tubulin isotypes: High-purity isotopically labelled equivalent peptides were purchased (AQUA peptides, Thermo Fischer Scientific, Bremen, Germany). For the SRM analyses, samples were submitted to a tube-gel sample preparation[45]. The gel pieces were reduced, alkylated and trypsin digested. The mixture of the heavy-labelled peptides and iRT peptides (Biognosys) were added to each sample prior to SRM analyses. LC-SRM analyses were performed on a Thermo TSQ Vantage (Thermo Scientific) coupled to a Dionex Ultimate 3000 MicroLC. The complete system was fully controlled by Xcalibur 2.1 (Thermo Scientific). Three to four transitions were monitored for both endogenous and heavy labeled peptides. Mass data collected during LC-SRM were processed with the Skyline open-source software[46]. Area intensity ratios of the heavy and the light forms of each peptide were manually checked. The concentration for target peptides of α4A- and α8-tubulins were normalized to the total α-tubulin content.

Western blotting: PVDF membranes were incubated with specific primary antibodies. The primary antibodies used were: monoclonal anti-α-tubulin (clone DM1a; Sigma Aldrich, Darmstadt, Germany) (1/1500), polyclonal anti-α4A-tubulin (pAb 7621) (1/250), anti tyrosinated α-tubulin (clone 1.A2, Sigma) (1/1000), and monoclonal anti-deTyr α-tubulin (clone 1D5) (0.4 µg/mL). Membranes were incubated with 50 ng/mL peroxidase-conjugated goat anti-rabbit or goat anti-mouse IgG (Jackson Immuno Research, PA, USA) and 50 ng/mL goat anti GAPDH peroxidase conjugated (Abcam, Paris, France) resolved by Clarity Western ECL Substrate (Bio-Rad, Hercules, CA, USA).

Immunofluorescence microscopy: Platelets fixed and captured on poly-Lysine slides were permeabilized for 5 min with 0.1% Triton X-100 in PBS and incubated sequentially for 30 min with 1 µg/mL α-tubulin mAb (clone DM1a, Sigma Aldrich) or tyrosinated α-tubulin mAb used at a 1/800 dilution (clone TUB.1A2, Sigma Aldrich) and 1 µg/mL GAM-Alexa 488 (Life Technologies, USA) in PBS containing 1% BSA. The platelets were washed thoroughly at each step with PBS. The slides were mounted in Mowiol (Mountant, Permafluor, Thermo Fisher Scientific, UK).

Transmission electron microscopy: MKs and washed platelets embedded in Epon were classically processed. The procedure we used to prepare the washed platelets was as

described in Cazenave et al.[24], and is well-established to preserve the platelets in their resting state. Transversal thin sections of the entire BM were cut, contrasted with uranyl acetate and lead citrate and examined under a CM120 TEM (FEI, Eindhoven, The Netherlands)[47]. MKs at stages I, II and III were counted manually on whole transversal sections as previously described[4]. To facilitate quantification, each square of the grids is defined as an area for examination (which equals 16,000 μm^2). The number of MTs was counted in individual platelets (n= 30-50 platelets) that were sectioned transversally and where they appear as a cluster of small circles.

Scanning electron microscopy: Platelets in suspension were fixed with 2.5% glutaraldehyde and allowed to adhere to poly-L-lysine-coated coverslips. Samples were dehydrated, air-dried, sputtered with platinum palladium, and examined at 10 kV under a PHENOM scanning electron microscope (PHENOM World, Eindhoven, The Netherlands).

MK culture:

- *Mouse:* Bone marrow cells were flushed from the femurs and tibias and passed successively through 21-, 23- and 25-gauge needles. Cells were then pelleted at 1,200 rpm for 7 min and resuspended at 1×10^8 cells/mL in PBS supplemented with 5% (v/v) rat serum and 2 mM EDTA to perform a Lin selection (Stem Cell Technologies). The Lin^- population was adjusted to 5×10^6 cells/mL in DMEM containing 2 mM L-glutamine, penicillin/streptomycin, 10% FBS and 50 ng/ml TPO with addition of 50 U/mL hirudin. Cultures were usually performed in 12-well tissue culture plates with 700 µl of cell suspension per well and incubated at 37°C under a 5% CO_2 atmosphere for up to 5 days.

- *Human:* $CD34^+$ cells seeded at 4×10^4 per mL were cultured in StemSpan SFEM medium supplemented with 20 ng/mL human LDL, CC220 (1X), and 1 µM StemRegenin-1 (SR1). On day 7, the cells in suspension were harvested, washed and cultured at 5×10^4/mL in StemSpan SFEM medium containing 50 ng/mL TPO for an additional 7 days. The percentage of MKs extending proplatelets was determined in the culture wells by phase-contrast microscopy. In each culture, at least 600 MKs were analyzed and images were acquired using a Zeiss Axio Vert.A1 microscope with a 20× objective (Marly le Roi, France).

Screening and gene sequencing of blood donors: DNA was extracted from buccal swabs using Maxwell 16 Blood Purification kit, amplified using AmpliSeq™ Library Kit 2.0

kit (ThermoFisher), and primers were digested with FuPa Reagent included in the kit. A barcode unique for each DNA was added to each library using the Ion Xpress™ Barcode Adapters kit (ThermoFisher) and library purification was performed using Agencourt® AMPure® XP Reagent magnetic beads (Beckman Coulter). Libraries were processed using Ion IC HiQ PI, kit (ThermoFisher) for sequencing on Ion Proton (ThermoFisher). Data was processed using Torrent Suite Software v5.2.1 and variants were annotated using SeqNext v4.0.1 (JSI) and also VCF files from the Torrent server (Variant caller 5.2). Variants were examined for their frequency in the normal population using the ExAC database.

Supplemental References:

45. Muller L, Fornecker L, Van Dorsselaer A, Cianferani S, Carapito C. Benchmarking sample preparation/digestion protocols reveals tube-gel being a fast and repeatable method for quantitative proteomics. *Proteomics.* 2016; 16(23): 2953–2961.

46. MacLean B, Tomazela DM, Shulman N, et al. Skyline: an open source document editor for creating and analyzing targeted proteomics experiments. *Bioinformatics.* 2010;26(7):966-968.

47. Eckly A, Strassel C, Cazenave JP, Lanza F, Leon C, Gachet C. Characterization of megakaryocyte development in the native bone marrow environment. *Methods Mol Biol.* 2012;788:175-192.
