# Supplementary material for: Correction: An essential role for α4A-tubulin in platelet biogenesis
Source: Life Sci Alliance. 2021 Jun 21;4(8):e202101132. doi: 10.26508/lsa.202101132 (PMC8321679; doi:10.26508/lsa.202101132)
Supplement: Reviewer comments [file LSA-2021-01132_review_history.pdf]

Correction : An essential role for α4A-tubulin in platelet biogenesis.

Francois Lanza, Catherine Strassel, Maria Magiera, Arnaud Dupuis, Morgane Batzenschlager, Agnes Hovasse, Irina Pleines, Paul Guéguen, Anita Eckly, Sylvie moog, Léa Mallo, Quentin Kimmerlin, Stephane Chappaz, Jean-Marc Strub, Natarajan Kathiresan, Henri de la Salle, Alain Van Dorsselaer, Claude Ferec, Jean-Yves Py, Christian Gachet, Christine Schaeffer, Benjamin Kile, and Carsten Janke
DOI: https://doi.org/10.26508/lsa.202101132

Corresponding author(s): Francois Lanza, EFS Alsace

Review Timeline:

Submission Date:	2021-06-11
Accepted:	2021-06-16

Scientific Editor: Eric Sawey, PhD

Transaction Report:

(Note: With the exception of the correction of typographical or spelling errors that could be a source of ambiguity, letters and reports are not edited. The original formatting of letters and referee reports may not be reflected in this compilation.)

June 16, 2021

RE: Life Science Alliance Manuscript #LSA-2021-01132

Dr. Francois Lanza
EFS Alsace
INSERM UMR S1255
10 rue Spielmann
Strasbourg F-67065
France

Dear Dr. Lanza,

Thank you for submitting your Correction entitled "Correction : An essential role for α4A-tubulin in platelet biogenesis.". This will now be prepared for publication in Life Science Alliance.

Sincerely,

Eric Sawey, PhD
Executive Editor
Life Science Alliance
http://www.lsajournal.org

June 16, 2021

RE: Life Science Alliance Manuscript #LSA-2021-01132

Dr. Francois Lanza
EFS Alsace
INSERM UMR S1255
10 rue Spielmann
Strasbourg F-67065
France

Dear Dr. Lanza,

Thank you for submitting your Correction entitled "Correction : An essential role for α4A-tubulin in platelet biogenesis.". This will now be prepared for publication in Life Science Alliance.

Sincerely,

Eric Sawey, PhD
Executive Editor
Life Science Alliance
http://www.lsajournal.org
